# Bioavailability of Oral Hydrocortisone Corrected for Binding Proteins and Measured by LC-MS/MS Using Serum Cortisol and Salivary Cortisone

**DOI:** 10.4172/jbb.1000365

**Published:** 2018

**Authors:** TN Johnson, MJ Whitaker, B Keevil, RJ Ross

**Affiliations:** 1 SimCyp, Sheffield, United Kingdom; 2 The University of Sheffield, Sheffield, UK; Faculty of Medicine, Dentistry and Health, United Kingdom; 3 Manchester Academic Health Science Centre (MAHSC), The University of Manchester, Core Technology Facility, 46 Grafton Street, Manchester, M13 9NT, United Kingdom

**Keywords:** Bioavailability, Hydrocortisone, Cortisol, Cortisone, Saliva

## Abstract

**Context:**

The assessment absolute bioavailability of oral hydrocortisone is complicated by its saturable binding to cortisol binding globulin (CBG). Previous assessment of bioavailability used a cortisol radioimmunoassay which has cross reactivity with other steroids. Salivary cortisone is a measure of free cortisol and LC-MS/MS is the gold standard method for measuring steroids. We here report the absolute bioavailability of hydrocortisone calculated using serum cortisol and salivary cortisone measured by LC-MS/MS.

**Methods:**

14 healthy male dexamethasone suppressed volunteers were administered 20 mg hydrocortisone either intravenously or orally by tablet. Samples of serum and saliva were taken and measured for cortisol and cortisone by LC-MS/MS. Serum cortisol was corrected for saturable binding using published data and pharmacokinetic parameters derived using the program WinNonlin.

**Results:**

The mean (95% CI) bioavailability of oral hydrocortisone calculated from serum cortisol, unbound serum cortisol and salivary cortisone was 1.00 (0.89-1.14); 0.88 (0.75-1.05); and 0.93 (0.83-1.05), respectively.

**Conclusion:**

The data confirm that, after oral administration, hydrocortisone is completely absorbed. The data derived from serum cortisol corrected for protein binding, and that from salivary cortisone, are similar supporting the concept that salivary cortisone reflects serum free cortisol levels and that salivary cortisone can be used as a non-invasive method for measuring the pharmacokinetics of hydrocortisone.

## Introduction

The assessment of bioavailability of oral hydrocortisone is complicated by its saturable binding in the therapeutic range to cortisol binding globulin (CBG). Ninety percent of serum cortisol circulates bound to cortisol binding globulin, 5% to generic binding proteins, such as albumin and α-1 glycoprotein, and only 5% is unbound or ‘free’ [1]. CBG has high affinity for cortisol but lower capacity whereas albumin has a lower affinity and higher capacity and Lentjes and Romijn published Data from which free unbound cortisol could be calculated [2]. An increase in the free fraction results in an increase in clearance with dose and therefore less than dose proportional increases in C_max_ and bioavailability as measured by area under the curve (AUC) [3]. The only previous study of hydrocortisone bioavailability reported absolute bioavailability to be 0.96 (CI 0.82 to 1.09) [3], but used a radioimmunoassay to measure cortisol. Immunoassays have seen shown to give variable results depending on the specificity and sensitivity of the antibody used and LC-MS/MS is now the gold standard method for measuring steroids [4].

The measurement of steroid hormones in saliva provides a non-invasive method for assessing free steroid. Free cortisol is converted to cortisone by 11β hydroxy-steroid dehydrogenase (11βHSD) type 2 which is highly expressed in salivary glands [5]. In serum, cortisol levels exceed cortisone and the ratio of cortisol to cortisone is approximately 4:1 [6]. Free cortisol diffuses into saliva where it is rapidly converted to cortisone as the salivary glands have a high level of 11βHSD type 2. Thus, in saliva, the ratio of cortisol to cortisone is reversed compared to serum, with >4:1 salivary cortisone to cortisol [6]. Salivary cortisol and cortisone both show a close relationship to serum cortisol and serum free cortisol [6,7]. Ninety-four percent of the variation in serum cortisol is predicted from changes in salivary cortisone, salivary cortisone shows a closer relationship to serum cortisol than salivary cortisol, salivary cortisone is detectable at low levels of serum cortisol and can be measured in saliva without interference from oral hydrocortisone [6].

Here we report, for the first time, absolute bioavailability of oral hydrocortisone measured using LC-MS/MS and a comparison of bioavailability derived from: total serum cortisol; calculated free serum cortisol; and salivary cortisone.

## Methods

Fourteen healthy male subjects with a median (IQR) age of 28 (25-36) yrs, weight 83 (75-90) kg and BMI 25.3 (23.1-26.3) kg/m^2^ participated in the study as previously reported [6]. The study was approved by the South-East Wales Research Ethics Committee and all the subjects have given written informed consent. Each subject was administered 20 mg oral hydrocortisone and 20 mg IV at 07:00 h in random order. Serum samples for cortisol and salivary samples for cortisol and cortisone were taken at – 10 min, 15 min, 30 min, 45 min, 60 min, 1 h 15 min, 1 h 30 min, 2 h, 2 h 30 min, 3 h, 4 h, 5 h, 6 h, 8 h, 10 h and 12 h. Subjects received dexamethasone 1 mg orally at ~2200 h on Day 1 and at ~0600 h and ~1200 h on Day 2 during each study period to suppress the hypothalamic-pituitary-adrenal axis. All volunteers were given a standard mixed meal on Day 1 at 1900 h and on Day 2 at 0800 h and 1300 h.

Assays: LC-MS/MS analysis for serum, free and salivary cortisol/cortisone was performed using a Waters Xevo TQ-MSTM mass spectrometer and a Waters AcquityTM LC system with an electrospray source operated in positive ionisation mode [8]. The lower limit of quantitation (LLOQ) for serum cortisol was 12.5 nmol/L. The inter-assay imprecision was 8%, 7% and 6% at concentrations of 80 nmol/L, 480 nmol/L and 842 nmol/L respectively. Salivary cortisol and cortisone were measured with a modified LC-MS/MS assay with lower limits of detection 0.80 nmol/L (salivary cortisol) and 0.50 nmol/L (salivary cortisone). Intra-assay coefficient of variation (CV)s were less than 9.3% and less than 7.9%; and inter-assay CVs were less than 9.7% and less than 10.3% at 1.8 nmol/L to 52.2 nmol/L of salivary cortisol and 3.6 nmol/L to 96 nmol/L of salivary cortisone, respectively [9].

## Data analysis

For the calculation of pharmacokinetic (PK) end-points, serum cortisol and salivary cortisol & cortisone concentrations below the limit of quantification (BLQ) were assigned a value of zero. Total serum cortisol and salivary cortisone concentrations following either IV or oral hydrocortisone were adjusted by subtracting the – 10-min (min) sample, any adjusted values that were less than zero were set to zero. Serum cortisol was corrected for saturable binding using published data [2] to give an estimate of unbound serum cortisol (note in the design of the original study no plans were made to measure unbound plasma cortisol). Briefly, this was achieved by extracting and plotting data on free fraction with plasma cortisol and deriving a polynomial function to describe the fraction unbound (fu) at different plasma cortisol concentrations. For each total plasma cortisol concentration, the corresponding fu was calculated and used to derive the unbound plasma cortisol concentration. The PK end-points, C_max_, AUC_0-inf_, T_max_ were derived from the individual total serum cortisol, unbound serum cortisol or salivary cortisone concentration-time data over 0-12 h using Non-compartmental analysis in WinNonlin Phoenix 32. In the case of a deviation from the theoretical time, the actual time of blood sample was used in the calculation. Any individual treatment profiles from subjects where the pre-dose cortisol demonstrates inadequate suppression were excluded.

Bioavailability for each individual was calculated from the IV and oral AUC_0-inf_ values using logarithmic transformation data, analysed using a fixed affects general linear model accounting for sequence and treatment. The geometric mean and 95% confidence interval around the geometric mean were calculated for bioavailability for each of total serum cortisol, unbound serum cortisol and salivary cortisone values.

## Results

**Figure 1:**
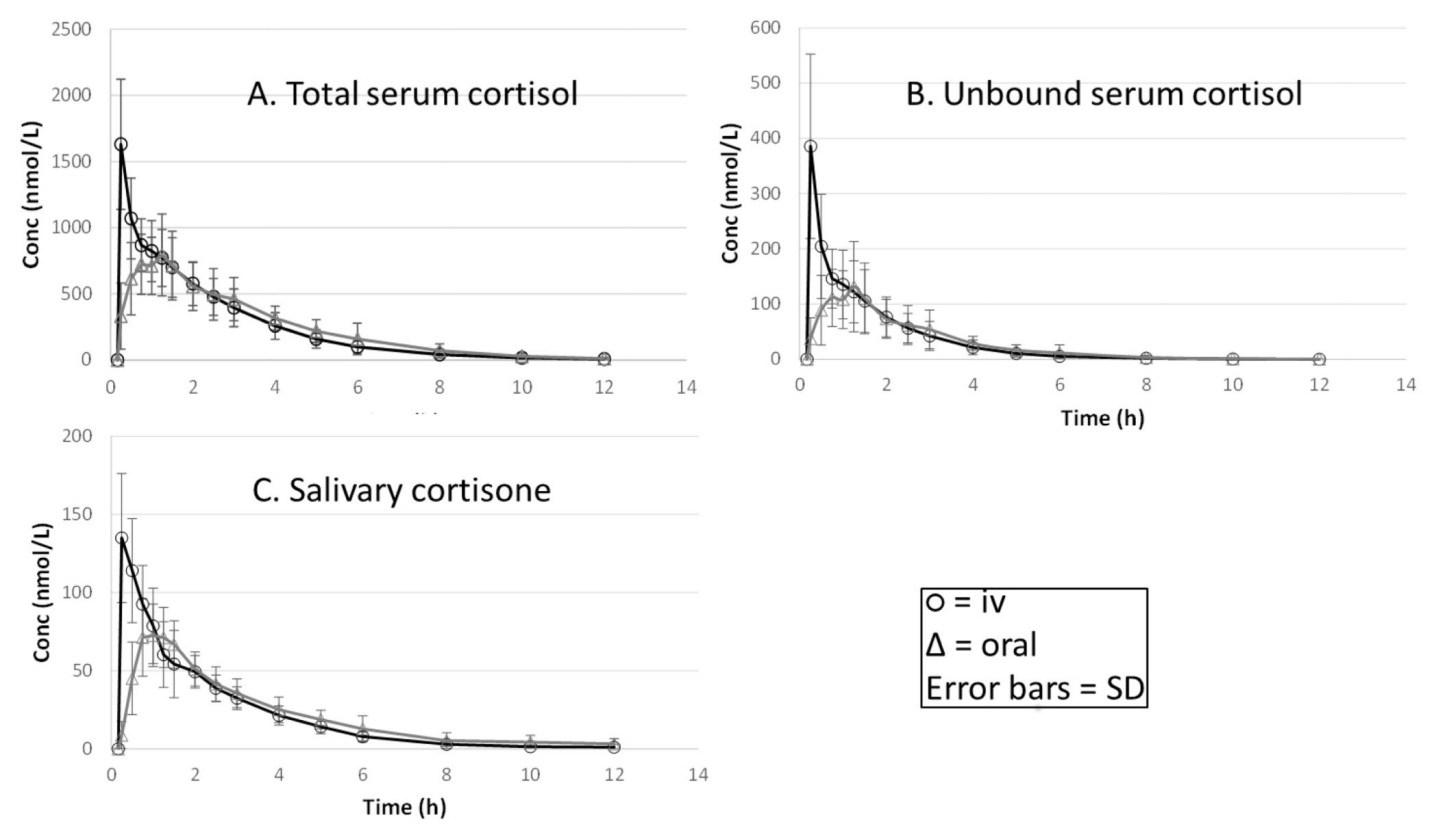
Pharmacokinetic figures for: **A.** Total serum cortisol; **B.** Calculated unbound serum cortisol; **C.** Salivary cortisone following oral or IV administration of 20 mg hydrocortisone.

The concentration-time profiles of serum cortisol, calculated unbound free serum cortisol and salivary cortisone are presented in the Figure 1 and the results from the pharmacokinetic analysis showing key parameters and variability in Table 1. The profiles for all three measures of cortisol were similar and the time of C_max_ was the same for salivary cortisone as for serum cortisol. As would be expected the C_max_ for IV is greater than that of oral hydrocortisone. Examining the variability in terms of CV% around each set of data in terms of AUC_0-inf_, most variability is seen for the calculated unbound serum cortisol (46% iv, 50% oral) and least for salivary cortisone (22% IV, 20% oral), similar results were seen for the C_max_. The absolute bioavailability of hydrocortisone was not statistically different for the three methods of measurement but for the calculated unbound and salivary cortisone the absolute bioavailability was <100% (88% and 93%, respectively) whereas for total serum cortisol it was 100%.

## Discussion

We have shown that oral hydrocortisone is completely absorbed with an absolute bioavailability of 100% when calculated from total serum cortisol and is not statistically different from 100% when calculated using unbound serum cortisol or salivary cortisone. The results using LC-MS/MS in our study confirm those previously published by Derendorf et al., who determined a bioavailability of 96 ± 20% using a radioimmunoassay [3]. The mean total plasma AUC of 1009 ± 383 ng/ml*h, C_max_ of 319 ± 109 ng/ml and median T_max_ of 1.125 h (0.5-1.5 h) from this study was similar to that reported by Derendorf et al of 1163 ng/ml*h, 305 ± 57 ng/ml, and 1.2 h (0.4-2 h) respectively, following a 20 mg oral dose. The results for C_max_ and T_max_ for 20 mg hydrocortisone are also in agreement with other previously published studies showing a range of 225-285 ng/ml after 1-1.4 h [10].

The absolute bioavailability was non-significantly lower for calculated unbound and salivary cortisone at 88 and 93%. This is not surprising, as due to non-linear and saturable protein binding, free cortisol levels will be higher immediately after IV administration compared to the oral route. This will potentially lead to higher total clearance and lower AUC_0-inf_ of the IV compared to the oral formulation especially at the early time points leading to an over-estimation of the bioavailability. This illustrates the potential problem of conducting bioavailability studies where there is non-linearity in the pharmacokinetics of a drug, these issues have been previously identified [11]. In the case of hydrocortisone either the unbound plasma cortisol or the salivary cortisone (as a surrogate for unbound plasma cortisol) concentrations will more likely give a more accurate assessment of absolute bioavailability compared to using total plasma cortisol concentrations. From our own observations, the differences in bioavailability for hydrocortisone do not seem to be significantly affected by the non-linear protein binding.

One limitation of the current study is that individual total serum cortisol concentration-time data were used to calculate the unbound cortisol profiles based on a literature derived model, errors introduced here may have contributed to the higher variability seen in the data. Ideally further research is needed where the actual unbound plasma cortisol is measured for each individual prior to data analysis and calculation of bioavailability. Interestingly least variability was observed in the salivary cortisone data suggesting that for this drug the data from this measure is reliable for calculating bioavailability. Another limitation is the 2 × 2 nature of this study design which may limit the detection of intra-individual variability and also detection of possible carry over effects [12].

## Conclusion

In conclusion, this paper demonstrates the use of alternative approaches in the determination hydrocortisone bioavailability that overcome the issue of non-linear pharmacokinetics of the drug due to non-linear protein binding. Atypical pharmacokinetics may influence the results when determining bioavailability and should thus be considered at the study design stage. The results confirm that salivary cortisone reflects unbound serum cortisol levels and demonstrate that salivary cortisone can be used clinically as a non-invasive method for measuring cortisol exposure after hydrocortisone administration. This is especially of value in monitoring children with adrenal insufficiency to check for appropriate hydrocortisone replacement [13].

## Figures and Tables

**Table 1:** Pharmacokinetic parameters derived from measurement of serum cortisol, calculated unbound serum cortisol and salivary cortisone following oral and iv administration of 20 mg hydrocortisone.

Route	Measure	AUC_0-inf_ (nmol/L.h) Mean ± SD)	C_max_ (nmol/L) Mean ± SD	T_max_ (h) Median and range	Bioavailability GeoMean and Cl GM
IV	Total serum cortisol	2900 ± 926	1629 ± 491		
Oral	Total serum cortisol	2779 ± 1058	880 ± 302	1.125 (0.5-1.5)	1.0 (0.89-1.14)
IV	Unbound serum cortisol	391 ± 182	386 ± 167		
Oral	Unbound serum cortisol	391 ± 165	153 ± 81	1.125 (0.5-1.5)	0.88 (0.75-1.05)
IV	Salivary Cortisone	258 ± 58	142 ± 22		
Oral	Salivary Cortisone	239 ± 48	88 ± 17	1.0 (0.75-1.5)	0.93 (0.83-1.05)
